# Cerebrospinal fluid biomarkers of infantile congenital hydrocephalus

**DOI:** 10.1371/journal.pone.0172353

**Published:** 2017-02-17

**Authors:** David D. Limbrick, Brandon Baksh, Clinton D. Morgan, Gakwaya Habiyaremye, James P. McAllister, Terrie E. Inder, Deanna Mercer, David M. Holtzman, Jennifer Strahle, Michael J. Wallendorf, Diego M. Morales

**Affiliations:** 1 Department of Neurological Surgery, Washington University in St. Louis, School of Medicine, Saint Louis, MO, United States of America; 2 Department of Pediatrics, Washington University in St. Louis, School of Medicine, Saint Louis, MO, United States of America; 3 Barrow Neurological Institute, Phoenix, AZ, United States of America; 4 Department of Pediatric, Brigham and Women’s Hospital, Harvard Medical School, Boston, MA, United States of America; 5 Department of Neurology, Washington University in St. Louis, School of Medicine, Saint Louis, MO, United States of America; 6 Department of Biostatistics, Washington University in Saint Louis, School of Medicine, Saint Louis, MO, United States of America; Medical Photonics Research Center, Hamamatsu University School of Medicine, JAPAN

## Abstract

**Introduction:**

Hydrocephalus is a complex neurological disorder with a pervasive impact on the central nervous system. Previous work has demonstrated derangements in the biochemical profile of cerebrospinal fluid (CSF) in hydrocephalus, particularly in infants and children, in whom neurodevelopment is progressing in parallel with concomitant neurological injury. The objective of this study was to examine the CSF of children with congenital hydrocephalus (CHC) to gain insight into the pathophysiology of hydrocephalus and identify candidate biomarkers of CHC with potential diagnostic and therapeutic value.

**Methods:**

CSF levels of amyloid precursor protein (APP) and derivative isoforms (sAPPα, sAPPβ, Aβ_42_), tau, phosphorylated tau (pTau), L1CAM, NCAM-1, aquaporin 4 (AQP4), and total protein (TP) were measured by ELISA in 20 children with CHC. Two comparative groups were included: age-matched controls and children with other neurological diseases. Demographic parameters, ventricular frontal-occipital horn ratio, associated brain malformations, genetic alterations, and surgical treatments were recorded. Logistic regression analysis and receiver operating characteristic curves were used to examine the association of each CSF protein with CHC.

**Results:**

CSF levels of APP, sAPPα, sAPPβ, Aβ_42_, tau, pTau, L1CAM, and NCAM-1 but not AQP4 or TP were increased in untreated CHC. CSF TP and normalized L1CAM levels were associated with FOR in CHC subjects, while normalized CSF tau levels were associated with FOR in control subjects. Predictive ability for CHC was strongest for sAPPα, especially in subjects ≤12 months of age (*p*<0.0001 and AUC = 0.99), followed by normalized sAPPβ (*p* = 0.0001, AUC = 0.95), tau, APP, and L1CAM. Among subjects ≤12 months, a normalized CSF sAPPα cut-point of 0.41 provided the best prediction of CHC (odds ratio = 528, sensitivity = 0.94, specificity = 0.97); these infants were 32 times more likely to have CHC.

**Conclusions:**

CSF proteins such as sAPPα and related proteins hold promise as biomarkers of CHC in infants and young children, and provide insight into the pathophysiology of CHC during this critical period in neurodevelopment.

## Introduction

Hydrocephalus is a debilitating neurological condition affecting approximately 1 in every 1000 children born in the United States [1]. While traditionally viewed as an imbalance in the production and absorption of cerebrospinal fluid (CSF), hydrocephalus is now recognized as a complex disease with a pervasive impact on the central nervous system [2, 3]. Hydrocephalus results in structural deformation, axonal stretch, ischemia, inflammation, and impaired precursor cell proliferation/migration among other pathophysiological processes [1, 4–6]. Extensive derangements in the biochemical profile of CSF are thus expected in the setting of hydrocephalus, particularly in infants and children, in whom neurodevelopment is progressing in parallel with concomitant neurological injury. Consequently, experimental analysis of CSF may provide unique insights into the pathophysiology of hydrocephalus and also offer the opportunity to identify candidate biomarkers of hydrocephalus with potential diagnostic and therapeutic value.

Our previous work in post-hemorrhagic hydrocephalus (PHH) of prematurity has shown alterations in CSF levels of amyloid precursor protein (APP), L1 cell adhesion molecule (L1CAM), neural cell adhesion molecule 1 (NCAM-1), and other protein mediators of neurodevelopment which normalize after initiation of PHH treatment [7]. Further, we have found that CSF APP levels, and to a lesser extent NCAM-1 and L1CAM, correlate with ventricular size and possibly intracranial pressure in PHH, responding in parallel with ventricular decompression [8]. With this foundational work in PHH in mind, the primary objective of the current study was to characterize the CSF levels of APP and related isoforms/cleaved products, L1CAM, NCAM-1, tau, phosphorylated tau (pTau), and aquaporin 4 (AQP4) in non-hemorrhagic, congenital hydrocephalus (CHC) in order to investigate the possibility of a larger relationship between these CSF proteins and hydrocephalus.

## Materials and methods

### Ethics statement

Approval from the Washington University (WU) Human Research Protection Office (#201203151, 201203126) was acquired prior to initiation of this study. Informed Consent (IC) procedures were in accordance with the approved WU HRPO parameters. Written IC was obtained where possible; however, verbal consent was permitted in cases where parents/guardians were unable to travel to Washington University/St. Louis Children’s Hospital. In all cases, a log was kept with subject ID and date and individual providing IC.

### Research subjects

All patients ≤18 years of age presenting to St. Louis Children’s Hospital/WU School of Medicine for evaluation and/or surgical management of untreated, non-hemorrhagic CHC from 2010–2014 were considered for recruitment. For inclusion, CHC subjects were required to have ventriculomegaly on cranial imaging (frontal-occipital ratio (FOR) ≥0.4) [9] and at least one of the following: head circumference >98^th^ percentile for corrected age; bulging fontanel or splaying of the cranial sutures; papilledema; refractory headache, vomiting, or lethargy without other identifiable cause; or upgaze paresis/palsy. Exclusion criteria included previous surgical treatment for hydrocephalus; history of central nervous system infection or neoplasm; history of open spina bifida; hydranencephaly; and PHH of prematurity. Subjects meeting inclusion/exclusion criteria underwent surgical management of hydrocephalus following routine clinical care pathways at St. Louis Children’s Hospital. For a detailed record of clinical, radiographic, and neurosurgical parameters from the patients recruited for CHC, please refer to Table 1.

**Table 1:** Clinical, radiographic, and neurosurgical parameters for the 20 congenital hydrocephalic CSF samples used throughout this study.

Race	Sex	Birthweight (grams)	Birth EGA (weeks)	Age at Sample (months)	Aqueduct Status	Associated Developmental Brain MalformationBrain Malformation?	Genetic Studies	Genetic Group Assignment	Specific Genetic Anomaly	Surgery Type	FOR
Caucasian	F	NA	27	5	Stenosis	None	CMA	1	N/A	VPS	0.49
Caucasian	M	3465	40	0	Stenosis	None	None	1	N/A	VPS	0.59
Black	M	3033	38	8	Patent	DWV	None	1	N/A	VPS	0.77
Caucasian	M	3200	41	0	Stenosis	None	None	1	N/A	VPS	0.62
Asian	M	3600	39	9	Patent	None	None	1	N/A	VPS	0.55
Caucasian	F	2500	40	11	Patent	None	None	1	N/A	VPS	0.55
Caucasian	F	2948	39	4	Stenosis	None	None	1	N/A	VPS	0.58
Caucasian	M	3095	37	0	Stenosis	pACC	CMA; L1CAM	2, 3	G847X (L1CAM)	VPS	0.89
Caucasian	M	2820	38	1	Stenosis	MCD, meningocele	CMA	2, 3	1q25.2	VPS	0.61
Caucasian	M	710	31	1	Stenosis	None	CMA	2	11q24.2	VSGS	0.48
Caucasian	M	4040	36	0	Stenosis	Rhombencephalosynapsis, MCD, pACC, cerebellar tonsillar ectopia	CMA	3	N/A	VPS	0.78
Caucasian	F	3300	34	0	Stenosis	MCD, syntelencephaly, cerebellar tonsillar ectopia	CMA	3	N/A	VPS	0.79
Caucasian	F	2115	33	13	Patent	None	None	3	N/A	VPS	0.42
Caucasian	M	3900	40	0	Stenosis	None	None	3	N/A	VPS	0.57
Caucasian	F	4180	39	0	Stenosis	Open lip schizencephaly versus in utero infarct	CMA	3	N/A	VPS	0.83
Caucasian	F	3345	36	147	Stenosis	None	None	3	N/A	ETV	0.45
Caucasian	M	2800	37	29	Patent	None	None	3	N/A	VPS	0.49
Other	F	3970	38	0	Stenosis	None	CMA	3	N/A	VPS	0.63
Caucasian	M	3350	39	0	Stenosis	None	CMA	3	N/A	VPS	0.6
Black	M	4337	39	4	Stenosis	Hypoplastic cerebellum, focal periventricular cystic dilation	CMA	3	N/A	VPS	0.72

Genetic group assignment categories: 1 = CHC with no known associations; 2 = CHC with known genetic alteration; 3 = CHC with developmental anomaly involving the CNS or non-CNS organ systems.

AS: aqueductal stenosis; CMA: chromosomal microarray; CNS: central nervous systems; DWV: Dandy Walker Variant; ETV: endoscopic third ventriculostomy; MCD: malformation of cortical development; pACC: partial agenesis of the corpus callosum; VPS: ventriculoperitoneal shunt; VSGS: ventriculosubgaleal shunt.

### Cerebrospinal fluid samples

A Neonatal CSF Repository was established at WU in 2008 (WU Human Research Protection Office #201101887 and #201203126). For the purposes of this study, CSF samples were acquired at the time of initial CHC treatment. This study was initiated before the introduction of endoscopic third ventriculostomy (ETV) at our institution; thus, 18 subjects underwent placement of ventriculoperitoneal shunts while one patient had an ETV. One other CSF sample was acquired from a patient born at 31 weeks estimated gestational age who underwent initial ventriculosubgaleal shunting. In all cases, once CSF was acquired, the sample was immediately transported on ice from the operating room to the WU Neonatal CSF Repository, where it was centrifuged at 2500 rpm for 6 minutes, and the supernatant was aliquoted into microcentrifuge tubes (500μl each) and stored at -80°C until experimental analysis. For comparative analysis, age matched control CSF samples were obtained from patients ≤18 years of age without known neurological disease (corroborated on neuro-imaging) undergoing lumbar puncture for evaluation for infection, where cytological and microbiological (and in some cases PCR) evaluation of the CSF was negative. Age matched lumbar CSF from infants and children with seizures or stroke, termed other neurological diseases (OND), was also analyzed for an additional comparator group.

### Measurement of candidate biomarkers

As described previously, enzyme-linked immunosorbent assays (ELISAs) were used to measure the CSF levels of each protein [7, 8]. The following commercially available ELISA kits were used: APP (R&D Systems, Minneapolis, MN; catalog #DY-850); soluble APPα (sAPPα) and soluble APPβ (sAPPβ) (IBL-International, Hamburg, Germany; catalog #27734 and 27732, respectively); amyloid-β_42_ (Aβ_42_), tau, and pTau (Fujirebio, Ghent, Belgium, catalog #80177, 80226, 80062, respectively); NCAM-1 (R&D Systems; Minneapolis, MN; catalog #DY-2408); L1CAM (DRG, Mountainside, NJ; catalog #EIA5074); AQP4 (USCN, Houston, TX, catalog #SEA582Hu). Each CSF sample was run in duplicate for each ELISA, and the 96-well plates were read at 450nm on a Versamax microplate reader (Molecular Devices, Sunnyvale, CA). The Pierce Bicinchoninic Acid protein assay kit (Thermo Scientific, Waltham, MA) was used to estimate the concentration of total protein (TP) in each CSF sample as previously described [8]). Where noted, the CSF levels of each specific protein analyzed (APP, Aβ_42_, sAPPα, sAPPβ, L1CAM, NCAM-1, tau, pTau, and AQP4) were normalized by total CSF protein to account for any non-specific changes in total CSF protein.

### Statistical analysis

Data were expressed using mean ± standard deviation. Associations between continuous factors were assessed using the Pearson correlation coefficient. Simple logistic regression was used to estimate predictive models of CHC with biomarkers. ANOVA with paired contrasts was used to compare means across groups. Odds ratio (OR), sensitivity, and specificity were calculated for candidate cut-points of normalized CSF proteins for predicting CHC. All data analyses were performed using SAS^®^ 9.3.

## Results

### Characteristics of study subjects

Data from 20 patients with untreated CHC were analyzed for this study (Table 1). Fifteen subjects had MRI evidence of aqueductal stenosis (AS); the remaining 5 subjects demonstrated patency of the cerebral aqueduct on MRI imaging. None had imaging findings suggestive of 4^th^ ventricular outlet obstruction. Seven subjects had radiologic evidence of associated developmental brain malformations (Table 1). Six subjects had comorbid craniofacial anomalies, six had developmental cardiopulmonary anomalies, and five subjects had comorbid genitourinary anomalies. Ten subjects had chromosomal microarrays (CMAs) or targeted gene testing, 3 of which subjects had identifiable genetic alterations (Table 1). The one subject with an L1CAM mutation (G847X) was excluded from the L1CAM CSF biomarker analysis.

### CSF total protein and ventricular size

**Fig 1:**
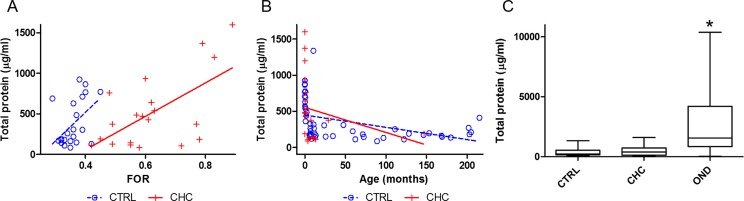
Relationship of CSF total protein with ventricular size, subject age, and subject grouping. A. Association of CSF total protein (TP) with ventricular size, estimated with frontal-occipital horn ratio (FOR) for control and congenital hydrocephalus (CHC) subjects. B. Association of TP with age for control and CHC subjects. C. Comparison of TP among control, CHC, and other neurological disorders (OND) groups. Note the positive correlation of TP with ventricular size (A) and the higher levels of TP in OND compared with control and CHC cases. *Denotes significance at *p*<0.05.

There was a positive correlation of CSF TP levels with ventricular size in CHC (n = 20, r = 0.613, *p* = 0.004) and in controls (n = 51, r = 0.48, *p* = 0.021) (Fig 1A and S1 Table). While CSF TP levels appeared to decrease with subject age, the association was not significant in CHC (n = 20, r = -0.252, *p* = 0.2847) but was significant in controls (n = 51, r = -0.43, *p* = 0.0017; Fig 1B and S2 Table). Interestingly, mean CSF TP levels were similar in control (356.55±277.6) and CHC groups (512.38±450.22) (*p* = 0.65) but higher among subjects with OND than among control and CHC subjects (*p* < 0.0001) (Fig 1C and Table 2).

**Table 2:** Cerebrospinal fluid levels of candidate biomarkers of congenital hydrocephalus. Mean ± SD CSF levels of candidate biomarkers of congenital hydrocephalus (both absolute and normalized by total protein) for control, congenital hydrocephalus (CHC), and other neurological disease (OND) groups, along with the *p*-value for the ANOVA with paired contrasts comparison among the groups.

	Control	CHC	OND	CHC—Control p-value	CHC—OND p-value	OND—Control p-value
**TP (ug/ml)**	356.55±277.6	512.4±450.2	2818±3113	0.6449	<0.0001	<0.0001
**APP (ng/ml)**	340.9±188.64	1117±957.1	328.3±166.52	<0.0001	<0.0001	0.9318
***Normalized APP***	1.31±0.93	2.59±1.64	0.3061±0.33	<0.0001	<0.0001	0.0027
**Abeta_42_ (ng/ml)**	0.461±0.37	1.101±0.79	0.430±0.59	<0.0001	0.0006	0.8479
***Normalized Abeta*_*42*_**	0.0015±0.001	0.0031±0.0022	0.00065±0.0013	<0.0001	<0.0001	0.0705
**sAPPα (ng/ml)**	114.95±97.83	701.1±768.9	77.5±54.43	0.0001	0.0017	0.8376
***Normalized sAPPα***	0.26±0.218	1.19±0.878	0.077±0.123	<0.0001	<0.0001	0.4042
**sAPPβ (ng/ml)**	325.1±281.34	3703±3304	250.3±187.25	<0.0001	<0.0001	0.9226
***Normalized sAPPβ***	0.826±0.798	8.741±11.13	0.257±0.395	0.0002	0.0026	0.8260
**L1CAM (ng/ml)**	19.57±20.35	182.7±239.0	10.28±16.39	<0.0001	0.0001	0.8017
***Normalized L1CAM***	0.055±0.054	0.282±0.23	0.01118±0.023	<0.0001	<0.0001	0.2543
**NCAM-1 (ng/ml)**	161.3±92.31	266.7±220.9	155.9±119.66	0.0048	0.0232	0.8971
***Normalized NCAM-1***	0.657±0.558	0.626±0.511	0.1594±0.217	0.8133	0.0100	0.0017
**Tau (ng/ml)**	2.081±2.813	10.33±7.607	4.632±8.255	<0.0001	0.0138	0.2277
***Normalized Tau***	0.0038±0.0033	0.0319±0.0368	0.0044±0.0118	<0.0001	0.0020	0.9362
**pTau (ng/ml)**	0.76±0.926	1.97±2.137	0.3553±0.303	0.0040	0.0060	0.4518
***Normalized pTau***	0.0014±0.0012	0.0044±0.0042	0.0002323±0.0004	0.0002	<0.0001	0.2024
**AQP4 (ng/ml)**	0.5436±0.566	1.126±1.845	0.3146±0.3062	0.1867	0.1383	0.6407
***Normalized AQP4***	0.0016±0.0015	0.0024±0.0037	0.00032±0.00053	0.3811	0.0742	0.2245

### Candidate CSF biomarkers of congenital hydrocephalus

**Fig 2:**
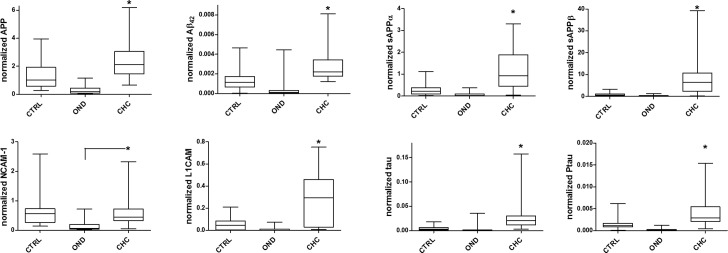
CSF biomarker levels in control, congenital hydrocephalus, and other neurological diseases. Box and whisker plots comparing normalized levels of CSF biomarkers measured in control, other neurological diseases (OND), and congenital hydrocephalus (CHC) subjects across all ages. Note the significant (*, *p*<0.05) increases in APP, Aβ_42_, sAPPα, sAPPβ, L1CAM, NCAM-1, tau, and pTau in CHC compared to both control and OND cases.

CSF levels of APP, sAPPα, sAPPβ, Aβ_42_, tau, pTau, L1CAM, and NCAM-1 but not AQP4 or TP were elevated in untreated CHC compared with age-matched controls or individuals with OND (Table 2). In order to account for any potential influence of TP on specific biomarker levels, each biomarker was normalized by CSF TP. With the exception of NCAM-1, CHC-associated elevations in biomarkers persisted after normalizing each CSF biomarker by TP (Table 2, Fig 2). Unless otherwise noted, CSF protein levels (APP, Aβ_42_, sAPPα, sAPPβ, L1CAM, NCAM-1, tau, and pTau) are reported throughout the remainder of this study as normalized by TP. With respect to ventricular size, normalized CSF tau levels were associated with FOR in control subjects only (R = 0.52; *p* = 0.038) while normalized CSF L1CAM demonstrated association with FOR in CHC subjects only (R = 0.55; *p* = 0.014) (S1 Table).

### Age-dependence of CSF biomarkers of congenital hydrocephalus

**Fig 3:**
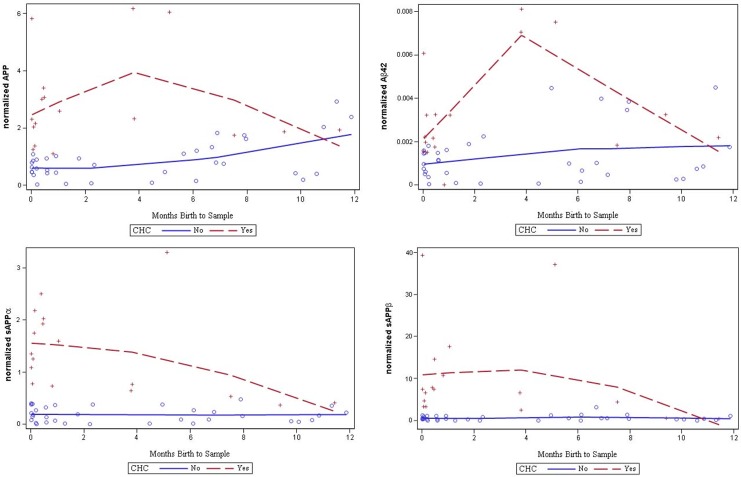
Relationship of CSF biomarker levels to congenital hydrocephalus and age at CSF sample. Regression analysis of normalized CSF APP, Aβ_42_, sAPPα, sAPPβ levels and age at sample (in months) in congenital hydrocephalus (CHC, shown in red) and non-CHC groups (control and other neurological diseases subjects, shown in blue). In general, APP, Aβ_42_, sAPPα, and sAPPβ levels were most different between CHC and control subjects <12 months of age.

**Fig 4:**
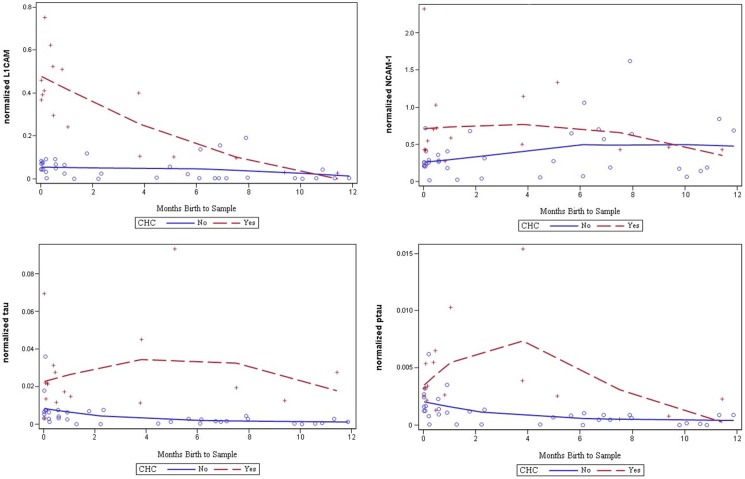
Relationship of CSF biomarker levels to congenital hydrocephalus and age at CSF sample. Regression analysis of normalized CSF L1CAM, NCAM-1, tau, and phosphorylated tau (pTau) levels and age at sample (in months) in congenital hydrocephalus (CHC, shown in red) and non-CHC groups (control and other neurological diseases subjects, shown in blue). Note that these proteins exhibited a similar pattern as APP and its isoforms by being most different in CHC <12 months of age.

Analysis of normalized CSF levels of APP, Aβ_42_, sAPPα, sAPPβ, L1CAM, NCAM-1, tau, and pTau across the entire cohort of CHC subjects, ranging in age between 0 and 215 months, demonstrated significant increases in CSF APP, Aβ_42_, sAPPα, sAPPβ, L1CAM, tau, and pTau in CHC compared with control and OND subjects (Fig 2, Table 2). Normalized CSF tau levels were associated with subject age alone in control subjects (R = -0.44; *p* = 0.009), while no other biomarkers were associated with age in control or CHC subjects (S2 Table). However, the observed differences in normalized CSF biomarker levels, including APP, Aβ_42_, sAPPα, sAPPβ, L1CAM, tau, and pTau, were greatest between CHC and control in subjects ≤12 months of age (Figs 3 and 4). Beyond 12 months, the separation became inconsistent. Given the complex relationship between CSF biomarkers and age, the remaining analyses in this study focus on CHC in infants ≤12 months of age.

### Predictive ability of CSF biomarkers of congenital hydrocephalus in infants ≤12 months of age

**Fig 5:**
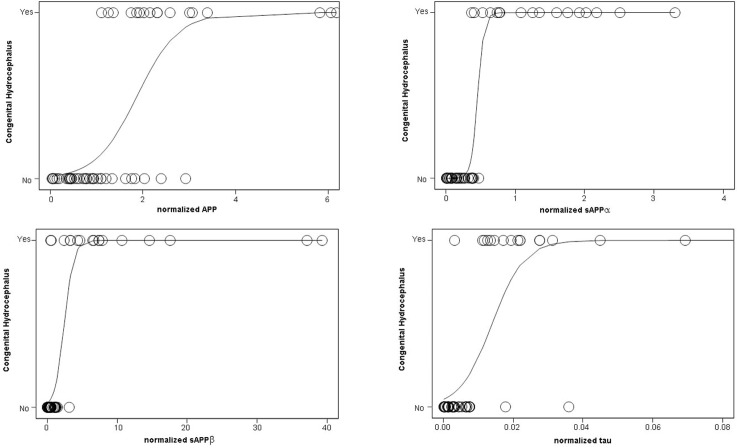
Logistic probability of CSF biomarkers for congenital hydrocephalus. Shown are logistic probability curves of normalized CSF levels of APP, sAPPα, sAPPβ, tau for congenital hydrocephalus (CHC) or no CHC (control and other neurological diseases subjects). Note that sAPPα, followed by sAPPβ, had the highest predictive ability for differentiating CHC.

**Fig 6:**
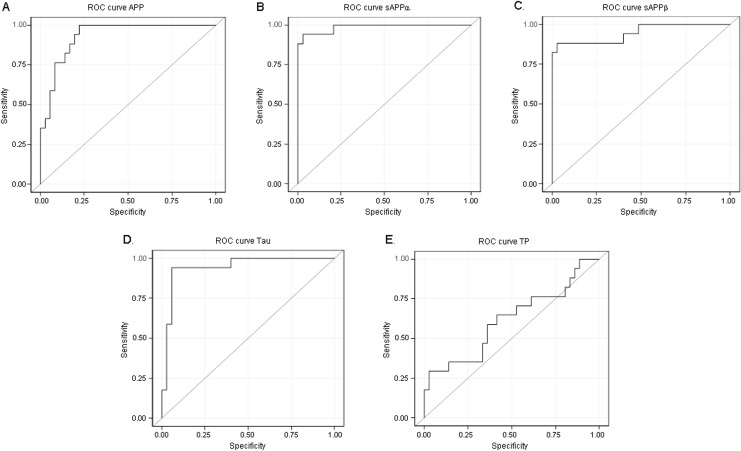
Receiver operating characteristic curves for CSF biomarkers of congenital hydrocephalus. Receiver operating characteristic (ROC) curves for normalized levels of APP, sAPPα, sAPPβ, tau, and CSF total protein (TP) in infants ≤ 12 months. Normalized sAPPα had the strongest predictive ability for congenital hydrocephalus (CHC), followed by normalized sAPPβ and tau; CSF TP alone showed no predictive ability for CHC (see also Table 4).

Higher CSF levels of APP, Aβ_42_, sAPPα, sAPPβ, L1CAM, tau, and pTau corresponded to greater incidence of CHC compared with control subjects and subjects with OND (Table 3, Figs 3–5). Among the specific CSF proteins examined, normalized sAPPα had the strongest predictive ability for CHC versus control (Table 4 and Fig 6, *p*<0.0001, area under the curve (AUC) = 0.99). When data from OND subjects were included, sAPPα remained effective in predicting CHC. Though not as strong, normalized sAPPβ, tau, APP, and L1CAM were also predictive for CHC over control and OND subjects (Table 4 and Fig 6). CSF TP alone showed no predictive ability for CHC (OR: 1.00, AUC: 0.61; Table 4 and Fig 6).

**Table 3:** Cerebrospinal fluid levels of candidate biomarkers of congenital hydrocephalus in infants ≤12 months of age. Mean ± SD CSF levels of candidate biomarkers of congenital hydrocephalus (both absolute and normalized by total protein) for CSF samples ≤12 months of age, for control, congenital hydrocephalus (CHC), and other neurological disease (OND) groups, along with the *p*-value for the ANOVA with paired contrast comparison among groups.

	Control	CHC	OND	CHC—Control p-value	CHC—OND p-value	OND—Control p-value
**TP (ug/ml)**	506.2±306.4	562.9±469.4	3597±3673	0.9039	<0.0001	<0.0001
**APP (ng/ml)**	409.7±197.55	1276±953.44	348.9±135.09	<0.0001	0.0002	0.7795
***Normalized APP***	1.05±0.658	2.834±1.64	0.257±0.273	<0.0001	<0.0001	0.0552
**Abeta42 (ng/ml)**	0.6747±0.389	1.232±0.789	0.5653±0.716	0.0047	0.0100	0.6415
***Normalized Abeta*_*42*_**	0.00159±0.0011	0.0033±0.0024	0.0007±0.0014	0.0016	0.0003	0.1578
**sAPPα (ng/ml)**	113.26±99.46	819.00±776.85	77.49±54.43	<0.0001	0.0002	0.8404
***Normalized sAPPα***	0.2227±0.132	1.365±0.827	0.077±0.123	<0.0001	<0.0001	0.4477
**sAPPβ (ng/ml)**	319.6±285.41	4348±3165.18	250.3±187.25	<0.0001	<0.0001	0.9221
***Normalized sAPPβ***	0.7288±0.629	10.24±11.45	0.257±0.395	<0.0001	0.0006	0.8533
**L1CAM (ng/ml)**	25.85±22.70	216.6±246.57	15.6±18.63	<0.0001	0.0010	0.8473
***Normalized L1CAM***	0.0544±0.051	0.3327±0.219	0.015±0.0278	<0.0001	<0.0001	0.4275
**NCAM-1 (ng/ml)**	171.1±63.28	307±215.27	195.7±126.05	0.0032	0.0608	0.6524
***Normalized NCAM-1***	0.4572±0.337	0.705±0.514	0.1598±0.225	0.0478	0.0014	0.0547
**Tau (ng/ml)**	2.70±3.035	10.84±7.552	4.632±8.255	<0.0001	0.0134	0.3988
***Normalized Tau***	0.00455±0.0036	0.0272±0.0228	0.0044±0.0118	<0.0001	0.0003	0.9849
**pTau (ng/ml)**	0.8747±0.9711	2.196±2.2356	0.355±0.303	0.0055	0.0036	0.3618
***Normalized pTau***	0.0015±0.0013	0.0042±0.0037	0.00026±0.0004	0.0005	0.0001	0.1757
**AQP4 (ng/ml)**	0.5436±0.5661	1.126±1.844	0.3146±0.3062	0.1867	0.1383	0.6407
***Normalized AQP4***	0.0016±0.0015	0.0024±0.0037	0.0003±0.0005	0.3811	0.0742	0.2245

**Table 4:** Evaluation of the predictive relationship between CSF biomarkers and congenital hydrocephalus. Logistic regression parameters for normalized levels of all potential biomarkers measured in this study are shown. The strongest predictors of CHC were normalized levels of sAPPα, sAPPβ, and Tau using cut points of 0.407, 2.43, and 0.0076 respectively. The weakest predictors of CHC were normalized pTau and NCAM-1. ROC AUC: receiver operating characteristics area under the curve; CI: confidence interval; Bonferroni corrected threshold (0.0055).

	Normalized APP	Normalized Aβ_42_	Normalized sAPPα	Normalized sAPPβ	Normalized L1CAM	Normalized Tau	Normalized pTau	Total Protein
**p-value**	<0.0001	0.0003	<0.0001	<0.0001	<0.0001	<0.0001	<0.0001	0.1069
**ROC AUC**	0.931	0.805	0.986	0.948	0.913	0.945	0.850	0.612
**Cut Point**	1.334	0.0015	0.407	2.43	0.095	0.0076	0.0021	900.0
**Odds Ratio (95% CI)**	31.07 5.72–168.5	34.91 4.10–297.49	528.0 30.99–8995.5	255.00 21.44–3033.3	56.00 9.17–342.13	170.67 16.42–1774.19	22.56 4.91–103.71	1.0833 0.28–4.18
**Relative Risk (95% CI)5% CI)**	10.57 2.68–41.61	14.81 2.12–103.68	32.00 4.62–221.48	16.87 4.36–65.28	13.22 3.37–51.88	27.79 3.99–193.35	7.47 2.45–22.77	1.06 0.42–2.68
**Sensitivity**	0.8824	0.9412	0.9412	0.8824	0.8750	0.9412	0.8235	0.7647
**Specificity**	0.8056	0.6857	0.9706	0.9714	0.8889	0.9143	0.8286	0.2500

Among subjects ≤12 months old in the CHC, control, and OND groups, a normalized sAPPα cut-point of 0.407 provided the best prediction of CHC (OR = 528, Sensitivity = 0.941, Specificity = 0.971, Table 4). Infants with sAPPα > 0.407 had 32 times greater risk (95% CI: 4.6–221) of having CHC than those with values below this cut-point. With a cut point of 2.43, normalized sAPPβ had a strong predictive association of CHC (OR = 255, Sensitivity = 0.88, Specificity = 0.97, Table 4) and infants above this cut point were almost 17 times more likely to have CHC. Normalized tau also demonstrated a high predictive value for CHC with a cut point of 0.0076 (OR = 171, Sensitivity = 0.941, Specificity = 0.914, Table 4). Infants with n-tau values above 0.0076 were at 28 times more likely to have CHC. Normalized L1CAM levels showed predictive ability, albeit somewhat less robust (OR = 56, Sensitivity = 0.88, Specificity = 0.89), with levels above the cut point of 0.095 associated with 13 times greater risk of CHC.

### Congenital hydrocephalus subgroup analyses

In order to further characterize CSF biomarker increases within CHC, subjects were analyzed for aqueduct of Sylvius status (AS or patent), the presence of developmental brain anomalies, or a known genetic alteration. No differences were seen among the three groups with respect to FOR or TP, and none of the CSF biomarkers demonstrated significant differences among groups (Table 5).

**Table 5:** Mean ± SD CSF levels of candidate biomarkers of CHC for CSF samples ≤12 months of age, for samples that showed aqueductal stenosis, CHC with developmental anomalies, or CHC with a known genetic alteration. All values shown are the normalized levels of proteins.

	CHC + Aqueduct Stenosis	CHC–Aqueduct Stenosis	CHC + Developmental Brain Malformation	CHC without Brain Malformation	CHC + Genetic Alteration	CHC–Genetic Alteration
FOR	0.642±0.132	0.604±0.101	0.648±0.154	0.600±0.106	0.660±0.209	0.606±0.121
TP (ug/ml)	608.1±480.0	339.6±305.5	627.0±519.8	443.8±382.7	929.1±603.4	455.2±380.7
APP	2.903±1.77	2.416±1.502	2.560±1.775	2.634±1.590	1.908±0.747	2.621±1.678
Abeta42	0.003±0.002	0.004±0.002	0.003±0.002	0.004±0.002	0.002±0.002	0.003±0.002
sAPPα	1.462±0.843	0.896±0.743	1.088±0.710	1.219+0.908	1.193±0.432	1.182±0.902
sAPPβ	11.25±11.84	3.62±2.89	8.066±10.91	8.569±9.70	10.97±4.28	8.95±11.39
L1CAM	0.371±0.207	0.232±0.225	0.325±0.237	0.257±0.198	0.375±0.189	0.282±0.233
NCAM-1	0.713±0.564	0.512±0.272	0.585±0.598	0.5678±0.320	0.431±0.155	0.636±0.523
Tau	0.027±0.024	0.039±0.046	0.032±0.043	0.035±0.042	0.015±0.002	0.033±0.038
pTau	0.005±0.004	0.005±0.006	0.005±0.004	0.005±0.005	0.006±0.004	0.004±0.004
AQP4	0.003±0.004	0.001±0.0005	0.003±0.004	0.001±0.0004	0.002±0.002	0.002±0.004

## Discussion

In the current study, levels of APP, sAPPα, sAPPβ, Aβ_42_, tau, pTau, L1CAM, NCAM-1, AQP4, and total protein were investigated in the CSF of children with and without congenital hydrocephalus. Of these candidate CSF biomarkers, all but CSF TP, AQP4, and NCAM-1 showed a robust association with CHC. Normalized sAPPα demonstrated a particularly strong relationship with infantile CHC, with high sensitivity and specificity. Soluble APPβ, tau, APP, and L1CAM were also able to discriminate CHC, though not as strongly as sAPPα. While CSF TP showed an association with ventricular size in controls and CHC, it did not exhibit predictive ability for CHC.

The pathophysiology of hydrocephalus is multifactorial with a pervasive impact on the brain. Previous studies of CSF composition in hydrocephalus (reviewed in [10–16] have suggested that periventricular axonal damage and demyelination [13, 17, 18], apoptosis [19], disruption of the blood-brain barrier [20], reduced cerebral blood flow accompanied by hypoxia and ischemia [14], altered metabolism [21], reductions in neurotransmitters and neuromodulators [18, 22–24], and neuroinflammation [13, 25–31] may all play important roles in the progression of hydrocephalus. Older studies of various biomarkers associated with these mechanisms in developing brains have suggested that none were robust enough to predict clinical outcome [10, 32], but more recent reports, especially those on adult and aging patients with idiopathic normal pressure hydrocephalus (iNPH), indicate that CSF levels of Aβ_42_ and tau, correlate well with clinical signs and symptoms [11, 12, 15, 18, 32–39]. Since APP must be delivered to synaptic membranes via axonal transport for cleavage into various isoforms [40, 41], and because periventricular white matter almost always exhibits impaired axonal transport and cytopathology during ventriculomegaly [2], it is understandable that CSF APP could originate from white matter axons and their terminal synapses. Experimental studies have shown that axoplasmic transport is impaired in hydrocephalus [42], synaptogenesis is modulated by APP[43, 44], and APP serves as a neurodevelopmental trophic factor [43]. Further, the processing of APP is highly regulated at multiple levels, and alterations in these pathways are etiologic in pathological conditions [45]. For example, cleavage of APP by α-secretase (e.g. ADAM10) releases sAPPα, whereas cleavage by the β-secretase β–site APP-cleaving enzyme (BACE-1) elaborates sAPPβ, yet only the latter is believed to be amyloidogenic. Any CHC-associated alteration of such balance could thus result in differential abundance of these isoforms in the CSF. The microtubule-associated protein tau stabilizes the axonal cytoskeleton [46]. Finally, the neuroepithelial cells of the ventricular zone and the ependymal lining of the ventricular wall rely on cell adhesion molecules such as L1CAM and NCAM for structural integrity, and ventricular zone junctional proteins are known to be impaired in hydrocephalus [47–49]. Therefore, while reduced CSF flow and protein flux in hydrocephalus could be responsible for our findings (discussed below), it is also possible that the changes we have observed in CSF biomarkers reflect pathological alterations in periventricular tissue as much as changes in CSF flow.

The CSF biomarkers selected for study in the current report have been previously examined in PHH of prematurity [7, 8] and represent a natural starting point for targeted investigation of CSF biomarkers in CHC. However, CSF alterations in CHC may vary significantly from other etiologies of hydrocephalus, including hemorrhagic, infectious, or other causes. Indeed, the classification of CHC itself may include a range of etiologies for hydrocephalus, each with different biological underpinnings. Seminal research studies reported over the last several years have implicated alterations in cell junction pathology [4, 48], precursor cell migration [49], ependymal polarity and cilia [50–53], and other mechanisms in the pathogenesis of hydrocephalus (reviewed in [1] and [6]). In a related project, we have used CSF proteomics to conduct a higher-level survey of pathophysiology at play in CHC; results from that effort are forthcoming.

Novel biomarkers of CHC and other forms of hydrocephalus are urgently needed to improve the clinical care and treatment of children affected by these conditions. At present, a diagnosis of hydrocephalus is usually made based on symptoms (e.g. irritability, nausea/vomiting, or headaches in a child able to convey symptoms), signs (e.g. tense fontanel, macrocephaly, papilledema, cranial nerve deficits), and ventricular enlargement on neuro-imaging. However, the symptoms and signs used in the diagnosis of hydrocephalus are nonspecific, and ventricular size may be affected by a number of conditions common to infants and children at risk for hydrocephalus, including brain malformations, intracranial hemorrhage, and hypoxia/ischemia. Clinically, the diagnosis of hydrocephalus and the optimal timing of initiation of treatment (CSF shunting or ETV) are often unclear. Once implemented, treatment is invariably challenging to assess, since imaging is frequently unreliable after shunting, and few other tools are available to provide insight into CSF shunt or ETV function or, importantly, optimizing the child’s neurodevelopmental trajectory.

While the most direct effect of these novel CSF biomarkers for CHC may be their diagnostic and therapeutic potential, the results reported herein also open new lines of scientific inquiry into the pathogenesis and pathophysiology of CHC. Elevations in CSF biomarkers, APP and tau in particular, may suggest recent or ongoing neurological injury, for example axonal stretch-related injury or synaptic disruption, as has been proposed in traumatic brain injury and other conditions (reviewed in [54]). In particular, Del Bigio and colleagues have repeatedly stressed the importance of periventricular white matter injury as a major factor in the pathophysiology of hydrocephalus [2, 55, 56]. Further, differential regulation of APP processing and amyloid isoforms may provide insight into specific pathways involved in the pathogenesis of hydrocephalus and its neurological sequelae and/or the repair mechanisms at play in neurological recovery. The finding of early elevations, but later normalization, of APP and other CSF biomarkers is in agreement with the notion of acute and chronic stages of hydrocephalus advanced by McAllister [6] and others[57, 58]. Experimental models have demonstrated an acute phase to hydrocephalus, in which there occurs an active pathophysiological insult and ensuing inflammation as well as a chronic phase in which ventriculomegaly stabilizes, the changes noted in the acute phase subside, and perhaps compensatory mechanisms become involved [57–59].

The relationships between increased CSF proteins and CSF physiology is likely to be complex. From a traditional perspective, obstructions that decrease CSF flow and/or absorption would be expected to impair clearance and cause CSF protein concentrations to rise. Fifteen of 20 of our patients exhibited aqueductal stenosis, potentially implicating CSF stasis in high TP and biomarker levels (though our limited analysis of 15 subjects with aqueductal stenosis versus 5 with patent aqueducts suggested no difference in TP or biomarker levels). In addition, an increase in total protein could have impacted CSF-parenchymal osmotic gradients and promoted ventriculomegaly in the same way that and dextrose- or sucrose-induced gradients cause mild-moderate ventriculomegaly in adult rats, dogs and cats [60–62]. Furthermore, clinical studies have shown that levels of surfactant proteins, specifically types A and C, increase significantly in hydrocephalus with and without aqueductal stenosis [63]. Surfactant protein A is associated with the blood-brain and blood-CSF barriers, while surfactant protein C is located in choroid plexus epithelial cells and ependyma. Thus, it is conceivable that CSF protein elevations, initially caused by periventricular tissue damage and/or CSF stasis, could exacerbate ventriculomegaly by impeding CSF flow and absorption as well as by drawing interstitial fluid from the parenchyma into the ventricles.

High CSF protein concentrations also could be related to alterations in the CSF glymphatic system (reviewed in Iliff et al [64–66]). This recently-described system helps mediate CSF absorption via paravascular pathways associated with both penetrating arterioles and microvessels within the cortical parenchyma. Impairment of these pathways during traumatic brain injury increases CSF levels of tau and this effect is exacerbated in mice lacking aquaporin-4 channels [67, 68]. Likewise, clearance of soluble amyloid-beta and intraventricular adeno-associated viruses is reduced in these knockout mice[69, 70]. While this mechanism of CSF absorption has not been well-studied in immature brains, aged rats with amyloid-beta injected into cortical parenchyma show diminished glymphatic/paravascular clearance[71]. Since aquaporin-4 channels are closely associated with the glymphatic system, it is worth noting that CSF aquaporin-4 levels were not changed in this limited study, making the role of glymphatic disturbance less certain.

In addition to PHH, many of the CSF biomarkers examined in the current report have been investigated in the setting of Alzheimer’s disease, Parkinson’s disease, and notably, iNPH, among other conditions (reviewed in[72]). For example, sAPPα and sAPPβ, but not tau and pTau, may also be useful in distinguishing iNPH from Alzheimer’s disease or possibly other conditions that affect this older population[33, 73–76]. Interestingly, we found previously that CSF sAPPα was an excellent predictor of PHH, though the sAPPα levels that we observed in PHH (lumbar CSF: mean 1667±1227 ng/ml, ventricular CSF mean 932.9±781.51 ng/ml) and in CHC in the current report (ventricular CSF, mean 701.1±768.9), are considerably higher than those described by Miyajima et al. for lumbar samples from patients with iNPH (152±60ng/ml), which were in their work decreased from levels in controls and Alzheimer’s patients. These differences may suggest very different pathophysiological processes in PHH, CHC, and iNPH but certainly suggest involvement of APP processing pathways in the pathogenesis of hydrocephalus more broadly.

A number of limitations must be acknowledged in this research project. While, to our knowledge, this study represents the largest study of CSF in non-hemorrhagic CHC, it nonetheless details an investigation of a modest sample size from a heterogeneous group of patients with likely myriad etiologies for hydrocephalus. The samples themselves span a wide range of ages with relatively fewer samples coming from individuals older than two years. Also contributing to the heterogeneity is the number and variability of associated brain abnormalities, non-CNS conditions, and genetic findings in the study population. It is likely that as we learn more about genetic alterations and their relationship to hydrocephalus that many of those individuals in this study in which no genetic etiology was identified will indeed have had a genetic cause and we were simply not yet able to detect it. While narrowing the scope of the project somewhat, subjects with hydrocephalus associated with myelomeningocele were deliberately excluded from the study, since factors such as active CSF leak or fluid shifts and uncertainty of infection presented challenging confounds to the current analysis. An inevitable limitation and challenge to studies of human CSF in children is the absence of true age-matched control CSF samples; the current study relies on CSF samples from human infants and children who underwent a lumbar puncture for clinical evaluation for sepsis or other diagnostic assessment where the CSF cultures and evaluation were negative. Ethical principles clearly prohibit routine CSF collection from asymptomatic patients. Another limitation inherent to this and similar studies is the potential for bias in cross-group comparisons introduced by rostrocaudal protein gradients, since control and OND CSF samples were acquired via lumbar puncture and CHC samples were acquired by ventricular cannulation. Reiber et al have demonstrated that different sources of CSF proteins exhibit different hydrodynamics, especially in relation to CSF flux variations associated with blood-brain and blood-CSF barriers [77–80]. For example, CSF tau clearly originates from cortical axons and oligodendrocytes even when measured in lumbar samples and is not dependent upon blood-CSF barrier dysfunction [81]. We recently addressed this bias in the setting of PHH of prematurity, where lumbar punctures are frequently performed early in the treatment of the condition, and CSF can be conveniently compared between PHH, control, and OND [82]. In PHH, differences observed in APP and derivative isoforms, L1CAM, and TP were found to be robust versus other conditions, even when controlling for CSF access site. Lumbar punctures are not commonly performed in infants and children with CHC, however, so a direct comparison is not possible. It is worth noting that our samples were not affected by the use of ventricular catheters because they were obtained prior to the initiation of surgical treatment. Likewise, intracranial pressure measurements are not commonly obtained in CHC patients, since patients are under general anesthesia at the time of surgery, and numerous factors (e.g. anesthetic agent, respiratory rate/pCO_2_, body position, fluid loss in dural opening or ventricular cannulation) impact intracranial pressure under these conditions. The relationship between intracranial pressure and CSF biomarkers is of great interest, however, and our basic science efforts are directed at delineating this relationship in an experimental model where such factors can be rigorously controlled.

## Conclusions

CSF biomarkers including APP, sAPPα, sAPPβ, tau, and L1CAM hold promise as biomarkers of CHC in infants and young children. Soluble APPα in particular demonstrated a strong relationship with infantile CHC, with high sensitivity and specificity. In addition to supporting potential diagnostic and therapeutic roles for the care of children with CHC, the results of this study provide insight into the pathophysiology of CHC during this critical period in neurodevelopment.

## Supporting information

S1 Table Relationship of CSF biomarkers to ventricular size. Pearson correlation coefficients (*R)* and corresponding *p*-values for normalized CSF biomarker levels and ventricular size (frontal-occipital horn ratio) across all study groups.
(PDF)

S2 Table Relationship of CSF biomarkers to age at CSF sample. Pearson correlation coefficients (*R)* and corresponding p-values for normalized CSF biomarker levels and age at CSF sample across all study groups.
(PDF)

## Abbreviations

APP: amyloid precursor protein
Aβ_42_: amyloid beta 42
AQP4: aquaporin-4
AS: aqueductal stenosis
AUC: area under the curve
CHC: congenital hydrocephalus
CMA: chromosomal microarray
CNS: central nervous system
CSF: cerebrospinal fluid
ELISA: enzyme-linked immunosorbent assay
ETV: endoscopic third ventriculostomy
FOR: frontal-occipital horn ratio
IC: informed consent
iNPH: idiopathic normal pressure hydrocephalus
L1CAM: L1 neural cell adhesion molecule
MRI: magnetic resonance imaging
NCAM-1: neural cell adhesion molecule-1
OND: other neurological disease
OR: odds ratio
PHH: post hemorrhagic hydrocephalus
pTau: phosphorylated tau
ROC: receiver operating characteristics
sAPPα: soluble APPα
sAPPβ: soluble APPβ
TP: total protein
WU: Washington University
