# Supplementary material for: Cerebrospinal fluid biomarkers of infantile congenital hydrocephalus
Source: PLoS One. 2017 Feb 17;12(2):e0172353. doi: 10.1371/journal.pone.0172353 (PMC5315300; doi:10.1371/journal.pone.0172353)
Supplement: S1 Table — Pearson correlation coefficients (R) and corresponding p-values for normalized CSF biomarker levels and ventricular size (frontal-occipital horn ratio) across all study groups. (PDF) [file pone.0172353.s001.pdf]

Supplemental Table 1. Relationship of CSF biomarkers to ventricular size. Pearson correlation coefficients (*R)* and corresponding *p*-values for normalized CSF biomarker levels and ventricular size (frontal-occipital horn ratio) across all study groups.

	Control		Congenital Hydrocephalus		Other Neurological Diseases	
	R	*p*-value	R	*p*-value	R	*p*-value
Total Protein	0.4798	0.0205	0.6133	0.0040	0.0610	0.8430
APP	-0.2373	0.2756	0.2197	0.3521	-0.3100	0.3026
Abeta42	-0.3965	0.0677	0.0100	0.9664	0.0858	0.7804
sAPPα	0.0738	0.8503	0.2027	0.3915	0.4388	0.2767
sAPPβ	-0.0863	0.8254	0.1101	0.6441	0.4534	0.2592
L1CAM	-0.2190	0.3154	0.5538	0.0139	-0.0870	0.7775
NCAM-1	-0.3640	0.0958	0.2198	0.3518	-0.2750	0.3632
Tau	0.5221	0.0380	-0.3402	0.1423	-0.2819	0.4988
pTau	0.1300	0.6720	-0.1585	0.5044	0.0010	0.9980
