# Supplementary material for: Cerebrospinal fluid biomarkers of infantile congenital hydrocephalus
Source: PLoS One. 2017 Feb 17;12(2):e0172353. doi: 10.1371/journal.pone.0172353 (PMC5315300; doi:10.1371/journal.pone.0172353)
Supplement: S2 Table — Pearson correlation coefficients (R) and corresponding p-values for normalized CSF biomarker levels and age at CSF sample across all study groups. (PDF) [file pone.0172353.s002.pdf]

Supplemental Table 2. Relationship of CSF biomarkers to age at CSF sample. Pearson correlation coefficients (*R)* and corresponding p-values for normalized CSF biomarker levels and age at CSF sample across all study groups.

	Control		Congenital Hydrocephalus		Other Neurological Disease	
	R	*p*-value	R	*p*-value	R	*p*-value
Total Protein	-0.4279	0.0017	-0.2516	0.2847	-0.1703	0.5605
APP	0.0546	0.7034	-0.2972	0.2031	0.0580	0.8439
Abeta42	-0.2567	0.0719	-0.2111	0.3718	-0.0847	0.7735
sAPPα	0.2947	0.1439	-0.4079	0.0742	0.0504	0.8976
sAPPβ	0.3042	0.1229	-0.2495	0.2888	0.1291	0.7406
L1CAM	-0.0636	0.6875	-0.4166	0.0760	-0.1879	0.5201
NCAM-1	0.2406	0.0924	-0.3216	0.1668	-0.0869	0.7676
Tau	-0.4368	0.0087	-0.1105	0.6430	-0.4347	0.2423
pTau	-0.1519	0.4147	-0.1485	0.5322	-0.4013	0.2504
